# 234. Phage Therapy with BX211 Demonstrates Positive Efficacy Results in Staphylococcus Aureus Diabetic Foot Osteomyelitis: Phase 2 Randomized, Double-Blind, Placebo-Controlled Clinical Trial

**DOI:** 10.1093/ofid/ofaf695.086

**Published:** 2026-01-11

**Authors:** Benjamin A Lipsky, Ariel Cohen, Siblian Boston, Christine Orlando, Mike Sowers, Joe Fackler, Rima Sandhu, Aravinda Vadlamudi, Rob cohen, Anantha Makineni, Edward Fang, Robert Hopkins, Jagoda Jablonska, David Zlotin, Ron Mordoch, Edith Kario, Jenia Gold, Myriam Golembo, Nitsan Halevy, Hila Sberro Livnat, Merav Bassan

**Affiliations:** University of Washington, Seattle, Washington; BiomX, Ness Ziona, HaMerkaz, Israel; BiomX Inc, Gaithersburg, Maryland; BiomX Inc, Gaithersburg, Maryland; BiomX Inc, Gaithersburg, Maryland; BiomX Inc, Gaithersburg, Maryland; BiomX Inc, Gaithersburg, Maryland; Adaptive Phage Therapeutics, Frederick, Maryland; BiomX Inc, Gaithersburg, Maryland; BiomX Inc, Gaithersburg, Maryland; BiomX Inc, Gaithersburg, Maryland; BiomX Inc, Gaithersburg, Maryland; BiomX, Ltd, Ness Ziona, HaMerkaz, Israel; BiomX Ltd, Ness Ziona, HaMerkaz, Israel; BiomX Ltd, Ness Ziona, HaMerkaz, Israel; BiomX, Ltd, Ness Ziona, HaMerkaz, Israel; BiomX, Ltd, Ness Ziona, HaMerkaz, Israel; BiomX, Ltd, Ness Ziona, HaMerkaz, Israel; BiomX Ltd, Ness Ziona, HaMerkaz, Israel; BiomX, Ltd, Ness Ziona, HaMerkaz, Israel; MB, Ness Zionna, HaMerkaz, Israel

## Abstract

**Background:**

*Staphylococcus aureus* (StA) is the most prevalent causative pathogen in diabetic foot osteomyelitis (DFO). Bacteriophage (phage) therapy offers a novel alternative or adjunct therapy to antibiotics, with potential to improve the frequently unsatisfactory outcomes of current standard-of-care. The objectives of this study were to assess the safety and efficacy of phage (BX211) in subjects with DFO colonized with StA.

**Figure:**
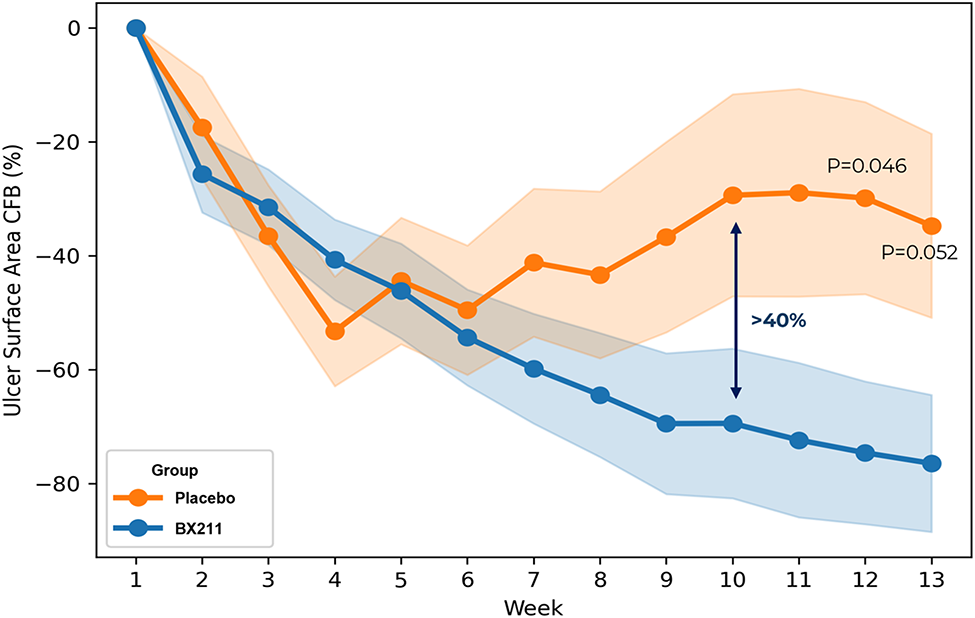
BX211 showed clinically relevant, statistically significant, reduction in ulcer surface area

Percent Area Reduction (PAR) from baseline of ulcer surface area (LS Mean ± SE).

Areas colored in orange and blue reflect the standard error. Full Analysis Set (FAS) population, all data are MMRM (Mixed Model Repeated Measure) LS mean and SEs (Standard Error). Statistical significance is not adjusted.

PAR – Percent Area Reduction, CI - Confidence Interval, CFB – Change From Baseline

**Figure:**
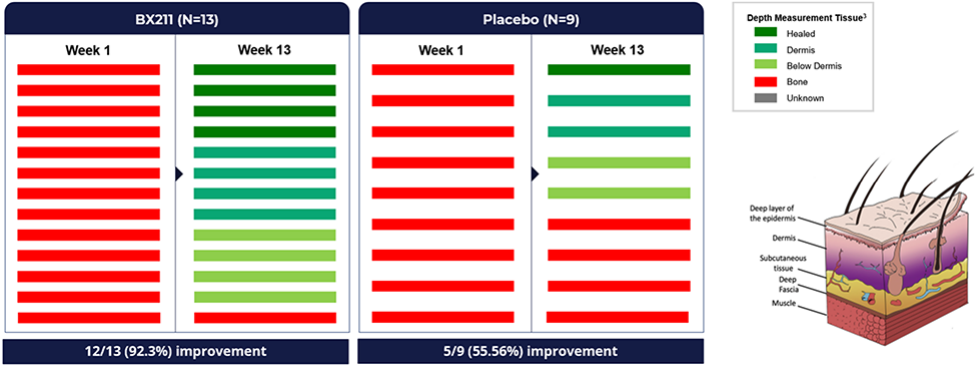
Patients with ulcers at bone depth displayed statistically significant better recovery in the BX211 group Change in tissue involvement of the ulcer for weeks 1 and 13.

For all patients at FAS (Full Analysis Set) population that had measured bone involvement at baseline and have a measured tissue involvement at week 13 as measured by a swab. The statistical test performed is Miettinen-Nurminen test, with p= 0.048, not adjusted.

In the figure, to avoid unblinding, Subcutaneous, Fascia and Muscle were placed in the same group.

**Methods:**

This phase 2, multicenter, randomized, double-blind, placebo-controlled trial investigated BX211 in 41 DFO patients with StA isolated from bone biopsy. BX211 is a phage therapy where phages from a pre-established "phage-bank" are individually matched to each patient's StA strain. Subjects were assigned (2:1) to an initial intravenous dose, followed by weekly topical doses of BX211 or placebo for 12 weeks, in addition to standard of care. Primary outcome was measured at week 13.

**Results:**

26 patients were randomized to BX211, 15 to placebo. BX211 demonstrated sustained and statistically significant^1^ greater Percent Area Reduction (PAR) of ulcer size (p = 0.046 at week 12 and 0.052 at week 13); separation from placebo started at week 7, and the difference was ≥ 40% by week 10 (Figure 1). Compared to placebo, BX211 also demonstrated statistically significant^1^ improvements in both ulcer depth at week 13 (in patients with ulcer depth defined as bone at baseline, ulcer depth was classified according to deepest tissue involved as measured by swab) (p=0.048) (Figure 2) and in limiting the expansion of ulcer area (p=0.017). Furthermore, the proportion of visits with no clinical evidence of infection (< 2 symptoms out of 5) through week 13 was notably higher in the BX211 group compared to placebo.

**Conclusion:**

BX211 therapy demonstrated sustained and statistically significant percent area reduction of ulcer size, compared to placebo, supported by additional improved clinical responses, and an overall positive safety profile. These favorable results warrant further studies of this promising therapy.

^1^p-values are non-adjusted

^2^This work was supported by Naval Medical Research Command (NMRC)-Naval Advanced Medical Development (NAMD) thru Medical Technology Enterprise Consortium (MTEC) by using an Other Transaction Authority (OTA).

**Disclosures:**

All Authors: No reported disclosures

